# Utilization of three-layers heterogeneous mammographic phantom through MCNPX code for breast and chest radiation dose levels at different diagnostic X-ray energies: A Monte Carlo simulation study

**DOI:** 10.3389/fpubh.2023.1136864

**Published:** 2023-03-03

**Authors:** Ghada ALMisned, Wiam Elshami, G. Kilic, Elaf Rabaa, Hesham M. H. Zakaly, Antoaneta Ene, H. O. Tekin

**Affiliations:** ^1^Department of Physics, College of Science, Princess Nourah Bint Abdulrahman University, Riyadh, Saudi Arabia; ^2^Medical Diagnostic Imaging Department, College of Health Sciences, University of Sharjah, Sharjah, United Arab Emirates; ^3^Faculty of Science, Department of Physics, Eskisehir Osmangazi University, Eskisehir, Türkiye; ^4^Institute of Physics and Technology, Ural Federal University, Yekaterinburg, Russia; ^5^Physics Department, Faculty of Science, Al-Azhar University, Asyut, Egypt; ^6^INPOLDE Research Center, Department of Chemistry, Physics and Environment, Faculty of Sciences and Environment, Dunarea de Jos University of Galati, Galaţi, Romania; ^7^Faculty of Engineering and Natural Sciences, Computer Engineering Department, Istinye University, Istanbul, Türkiye

**Keywords:** mammography, breast dosimetry, Monte Carlo (MC), MCNPX, X-ray

## Abstract

**Introduction:**

We report the breast and chest radiation dose assessment for mammographic examinations using a three-layer heterogeneous breast phantom through the MCNPX Monte Carlo code.

**Methods:**

A three-layer heterogeneous phantom along with compression plates and X-ray source are modeled. The validation of the simulation code is obtained using the data of AAPM TG-195 report. Deposited energy amount as a function of increasing source energy is calculated over a wide energy range. The behavioral changes in X-ray absorption as well as transmission are examined using the F6 Tally Mesh extension of MCNPX code. Moreover, deposited energy amount is calculated for modeled body phantom in the same energy range.

**Results and discussions:**

The diverse distribution of glands has a significant impact on the quantity of energy received by the various breast layers. In layers with a low glandular ratio, low-energy primary X-ray penetrability is highest. In response to an increase in energy, the absorption in layers with a low glandular ratio decreased. This results in the X-rays releasing their energy in the bottom layers. Additionally, the increase in energy increases the quantity of energy absorbed by the tissues around the breast.

## 1. Introduction

Mammography plays major role in diagnosing and assessing breast cancer. However, the advantages of mammography do not always outweigh the disadvantages. Some literature were concerned about the increase in the incidence of breast cancer after the start of the mammography screening programs (1). Due to the nature of the breast tissue, mammography X-ray tubes are manufactured with special anode/target materials and focal spot size. Mammography uses low tube voltage (25–30 kVp), low mA, longer exposure time and different filter combination to increase contrast difference as breast is composed of soft tissue. The use of the parameters leads to an increase in the radiation absorbed by the breast tissue due to photoelectric absorption and Compton scattering. With all advantages of screening mammography, it has been reported that the sensitivity of mammography decreases with the increase in breast density. Assessment of breast dose measurement is an essential aspect of radiation protection since screening mammography is performed periodically. The glandular tissue in the breast is the most tissue prone to radiation-induced mutations (2). Therefore, the Mean glandular dose (MGD) is the standard quantity used for radiation dosimetry in mammography as recommended by the ICRP in 1987. Radiation dose optimization is required to avoid increase in the MGD which might lead to increase of the breast cancer (3). Of course, direct measurement of the MGD is impossible, so conversion factors are used to relate measurable dose quantities to the MGD. Conversion factors are usually based on the characteristics of the breast (size and composition) and the x-ray spectrum used for mammography examination. Monte Carlo simulations were used to overcome the constraints of using phantoms to simulate breast composition and x-ray spectrum. Simple and complex geometrical models were developed to calculate conversion factors which relate the measurable quantity, the incident air kerma in mGy, to the MGD (4). In mammography, various Monte Carlo codes were used, including the MCNP or MCNPX (5, 6) and Geant4 (7). Notwithstanding, data on the density and composition of breast tissues are still minimal (4), and there is a need to estimate the energy imparted to the glandular tissues within the breast (8–10). The current study aims to perform a comprehensive study on establishing a detailed Monte Carlo simulation setup for different breast thicknesses and estimate MGD for these modeled breast phantoms. In contrast, the literature presents several models and concepts which would advance the simulation level of breast dosimetry techniques. Recently, Chang et al., (11) have proposed a three-layered homogeneous breast phantom for Monte Carlo simulation of normalized glandular dose coefficients in mammography. They described the dosimetry characteristics of this three-layer heterogeneous phantom with success. However, the absorbed energy ratios of these layers and the degree to which these ratios are dependent on layer properties remain unknown. With this momentum, we sought answers to a number of concerns that could provide motivation for further scientific community investigations, as well as to expand upon the findings of the first phase study by investigating the characteristics of this new three-layer heterogeneous model in deeper level. The results may be used for advanced assessment of MGD values during the patient risk assessment for mammography patients. The result can also be used for cancer risk assessment of those patients with existing cancer story or genetically tendency for some other cancer types.

## 2. Materials and methods

### 2.1. Monte Carlo simulations

**Figure 1:**
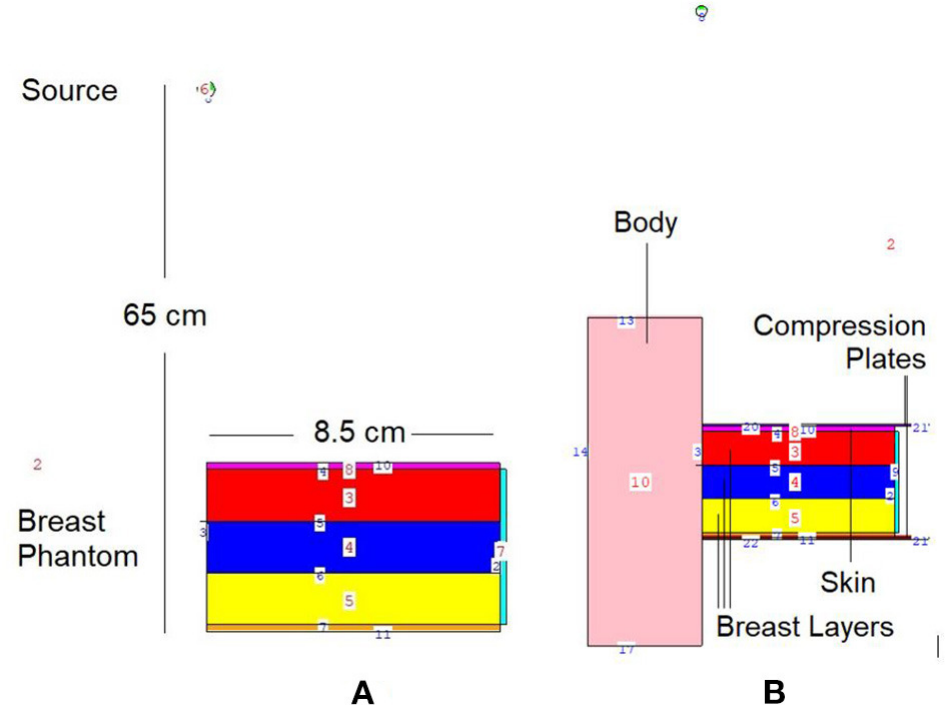
2-D view of modeled MCNPX simulation setup from **(A)** breast phantom **(B)** breast phantom with body phantom and compression plates (*via* MCNPX Visual Editor X22S).

**Figure 2:**
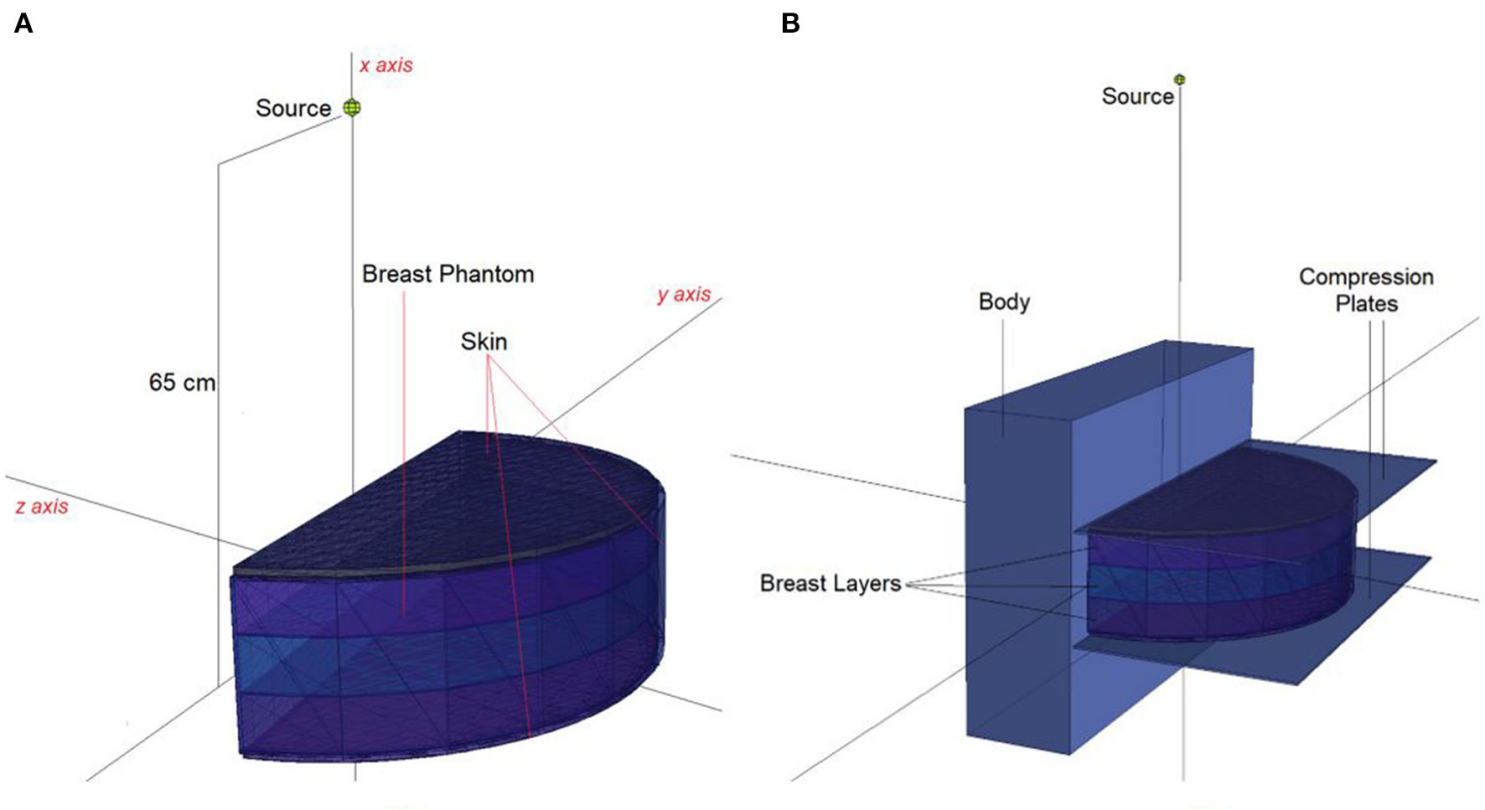
3-D view of modeled MCNPX simulation setup from **(A)** breast phantom **(B)** breast phantom with body phantom and compression plates (*via* MCNPX Visual Editor X22S).

In situations when experimental and clinical research is difficult or physically impossible to conduct, Monte Carlo simulation approaches may be used. This situation may range from organ dosimetry (12–15) in clinical research to shielding calculations (16–18) in radiation protection research, covering a broad spectrum. Some well-known Monte Carlo simulation-based radiation transportation codes such as MCNP (19), GEANT4 (20), EGSnrc (21), and FLUKA (22), have been utilized for such purposes. The geometric design and all simulation processes of the breast fandom used in the study were carried out with version 2.7.0 of MCNPX (19), which is a well-known and general-purpose Monte Carlo code. First, a three-layer breast phantom was constructed. For each layer, a unique Glandular Fraction value was assigned. The GF values of the layers were calculated in line with a previously researched geometric concept. In the MCNPX input file, each layer was specified as a separate CELL. The contents of these CELL volumes, as well as their elemental percentage fractions and densities, were specified in the INPUT file in accordance with the various GF values. In addition to their elemental percentages and densities, the skin layers surrounding the three-layer breast model's five surfaces were also defined. On the back of the designed breast model, a body phantom with the density of human tissue was added into the input file. In the last phase, a source with a source-image receptor distance of 65 cm was defined just on breast phantom. Figure 1 illustrates the breast phantom and source from a lateral view. As shown in Figure 1, the breast phantom is composed, from top to bottom, of cells 3, 4, and 5. The geometries of cells 8, 7, and 11 are carefully modeled from the lateral edge to the skin layer. Figure 1B depicts the cellular structure of cell 10 of the model body phantom. In addition, Figure 1B depicts lateral views of the compression plates placed on the top and lower sides of the breast phantom. After completing the input file, the modeled geometry was evaluated in 3D through using MCNPX image editor as well as any geometric mistakes were verified. Figure 2 displays the 3D geometries derived through the MCNPX visual editor (VE X22S) for the 2D geometries shown in Figure 1. As illustrated in Figure 2, the breast phantom is surrounded by compression plates in a way suited for clinical application. Meanwhile, the simulation studies were conducted through LENOVO ThinkStation P620 Tower Workstation with a processor of AMD Ryzen™ Threadripper.

### 2.2. Validation of MCNPX code

**Figure 3:**
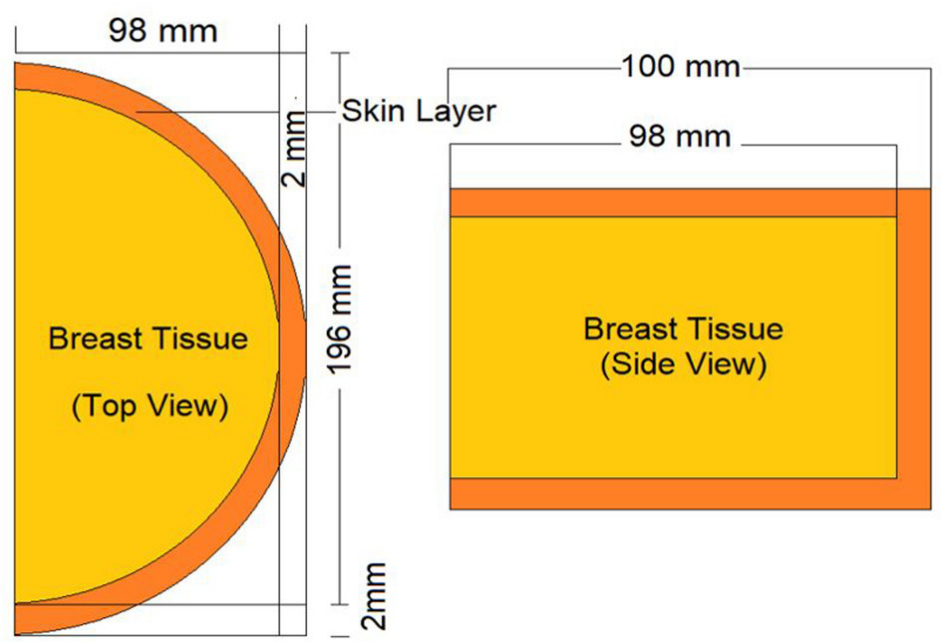
Appearance of modeled standard breast phantom in AAPM TG-195 report for validation phase of MCNPX.

For the validation process of the MCNPX code, the dosimeter guideline for the mammographic procedures specified in the previously published AAPM TG-195 (23) report was used. For this procedure, the manual's breast phantom was modeled using the same parameters (see Figure 3), and 80–20% adipose/glandular tissue was defined. Subsequently, the energy deposited in the modeled phantom was measured in accordance with the energy value specified in the guide (23). This amount (MeV/g) was calculated using the F6 (16, 19) tally mesh extension of the MCNPX code. Depending on the nature of the investigation and the anticipated result of photon-matter interaction, the MCNPX code employs a variety of tally meshes. Depending on the anticipated results of the investigation, each of them yields an unique outcome. The F6 Tally Mesh provides the amount of energy deposited per mass, often known as MeV/g. In light of our research objectives, we aimed to observe the quantity of energy deposited into the breast phantom's layers as a function of source energy. Therefore, it was appropriate to use F6 tally Mesh, which has been independently specified in there-layers, and at the end of the simulation, we acquired the deposited energy quantity for each layer. As the median value, the average of 4,754 eV/photon was determined after three iterations of counting. The observed deviation rate was below 0.3%. This minimal deviation rate was seen as an essential signal of the dependability of the data libraries and physics lists employed in this simulation investigation.

## 3. Results and discussions

**Figure 4:**
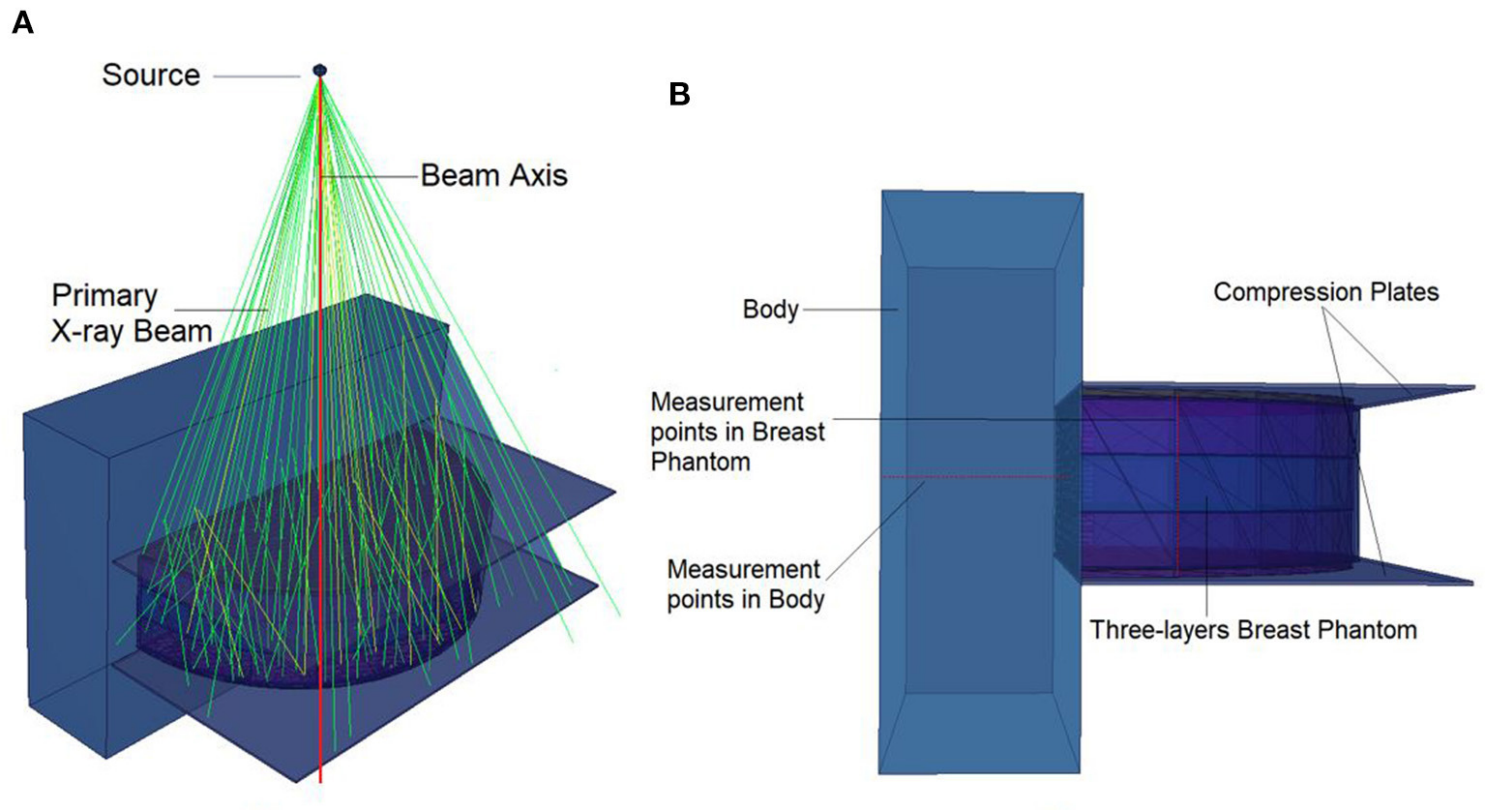
**(A)** Appearance of X-ray source. **(B)** Lateral view for measurement points.

This study's objective was to investigate the amount of absorbed energy, which occurs in the 3-layers heterogeneous breast model given in the literature and the behavioral changes in different layers. In the INPUT file, each layer of the modeled breast phantom was therefore characterized with a different cellular structure and composition (see Table 1). In line with the purpose of the study, the source was operated three times for each energy value in the low energy range of 26–36 keV with a track number of 10^8^ NPS. Each run operation conducted in the 26–26 keV energy range was recorded individually, and the mean results were recorded. Figure 4A depicts the exposure of a heterogeneous three-layer breast phantom between compression plates to X-rays generated by the source. Figure 4B depicts, from a lateral perspective, the measurement axes that are considered in the study of the energy-dependent change of the amount of absorbed energy in the layers and the energy-dependent change of the amount of absorbed energy in the body phantom that serve as the two main focal points of the current investigation. Three different F6 Tally outcomes were obtained from the OUTPUT file for three different layers modeled as separate cellular zones (see Figure 1). This was achieved by defining the desired outputs from the code separately according to the cells, in the section where the tally mesh definition is made in the data card section of the INPUT file. The code lines below are the desired absorbed energy amount lines for cells 3, 4, and 5 of the F6 tally meshes defined in the INPUT file.

F6:p 3.
F16:p 4.
F26:p 5.

**Table 1:** Elemental properties and densities of the modeled breast layers (11).

		**Weight percentage (%)**			
**Tissue**	**Density (g/cm^3^)**	***H***	***C***	***N***	***O***
GF Tissue (25%)	0.955	11	51	2.1	35.7
GF Tissue (50%)	0.982	10.7	40.1	2.5	46.4
GF Tissue (75%)	1.010	10.5	29.3	2.9	57
Skin	1.090	9.8	17.8	5	66.7

**Figure 5:**
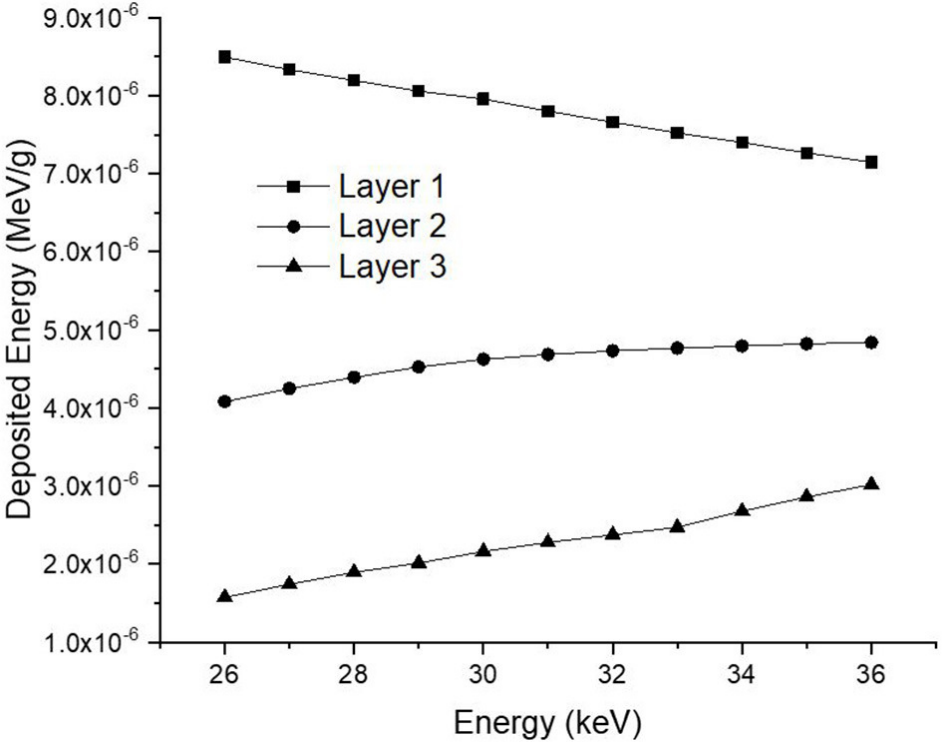
Variation of deposited energy (MeV/g) as a function of increasing energy (keV) for different breast layers.

**Figure 6:**
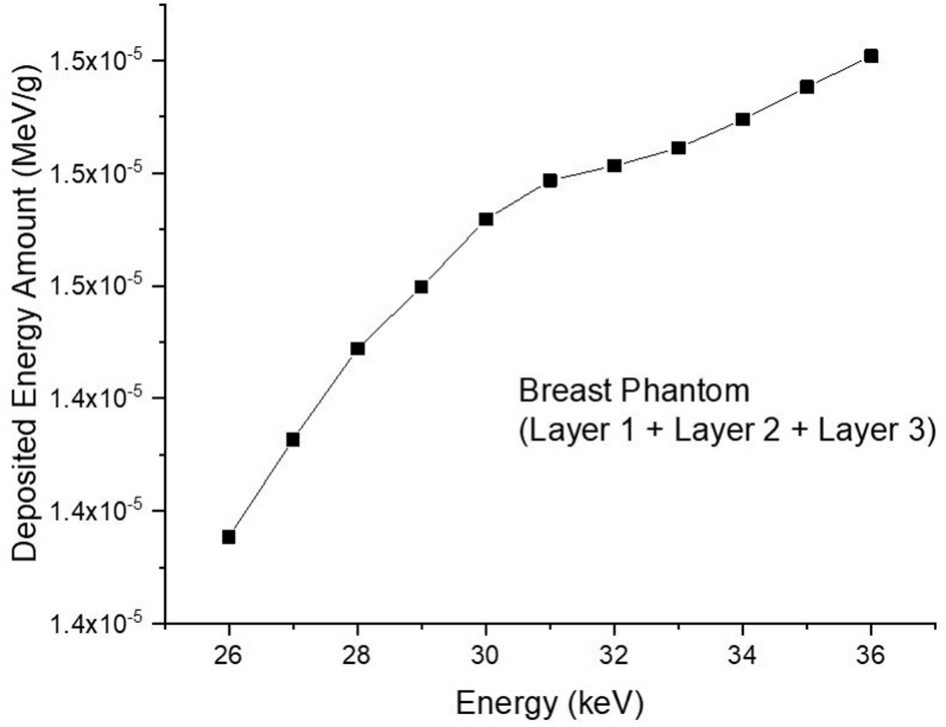
Variation of total deposited energy (MeV/g) as a function of increasing energy (keV).

**Figure 7:**
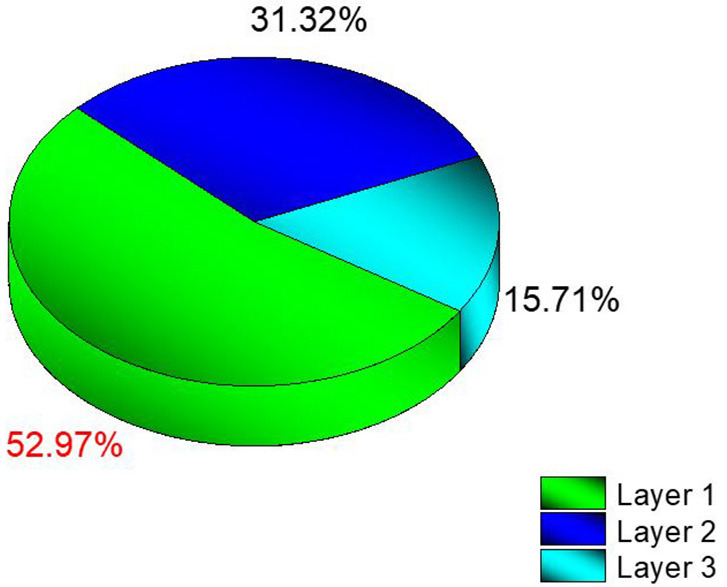
Average deposited energy amount (%) in different breast layers.

**Figure 8:**
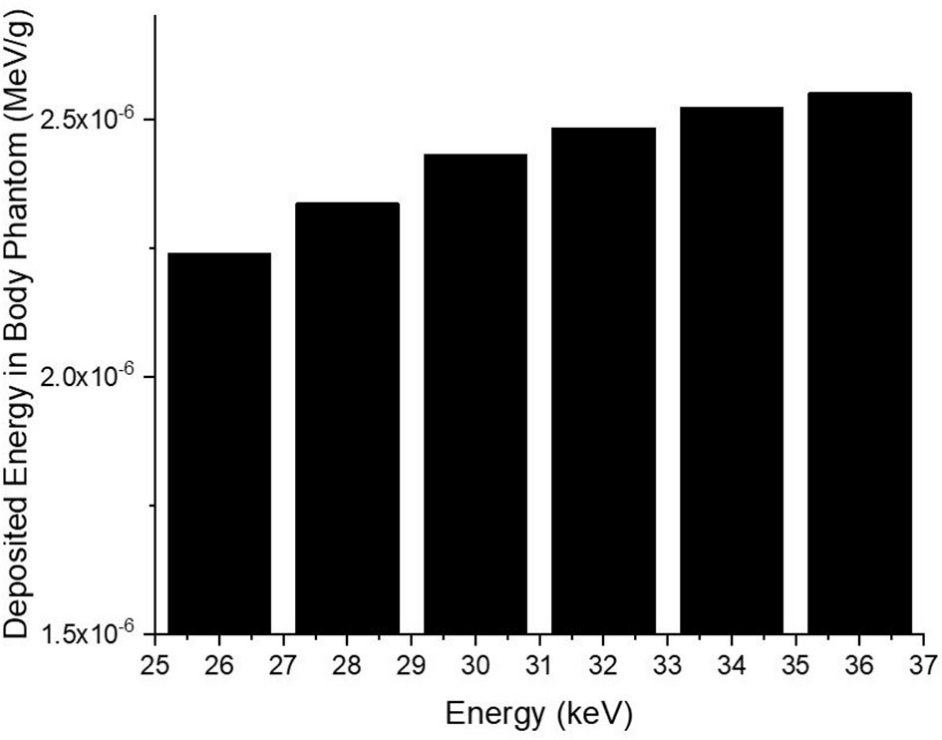
Variation of deposited energy amount (%) in modeled body phantom.

Consequently, the data acquired from the OUTPUT file were recorded individually for each energy value, and the quantity of energy absorbed in three distinct layers was analyzed as a function of increasing source energy. Figure 5 represents the amount of energy absorbed in the three different layers as a function of increasing source energy. As can be seen from Figure 1, the top layer closest to the source is the 3^rd^ cell and it is defined as Layer 1 in the INPUT file. The other layers are Cell 4 (Layer 2) and Cell 5 (Layer 3), respectively. As can be seen from Figure 5, the amount of energy absorbed in Layer 1 is at the maximum level at the lowest energy value of 26 keV. Along with this, the amount of energy absorbed in Layer 2 and Layer 3 decreased, respectively. Low-energy 26 keV photons penetrate the first layer after their first interaction with the skin layer, therefore releasing most of their energy into Layer 1. This is a natural consequence of the X-ray penetrating capabilities (24). However, the increase in energy from 26 keV to 27 keV caused a decrease in the quantity of energy absorbed by the first layer. Parallel to this, the quantity of energy absorbed by the second and third layers increased. This is due to the shift of X-rays, whose penetrating characteristics rise from 26 keV to 27 keV, to the second layer, where they are absorbed more than in the first layer. Reduced absorption in the first layer allows these unabsorbed X-rays to get through to the second layer (25, 26). Therefore, the unabsorbed X-rays have not released their energy in the first layer. This unreleased energy has been transmitted through first layer, absorbed by other layers, and has increased the quantity of energy absorbed by successive layers such as Layer 2 and Layer 3. Figure 5 depicts this occurrence as a decrease in Layer-1 and an increase in Layers 2 and 3 as a result of the rise from 26 keV to 36 keV. The layer with the lowest density among the three distinct layers specified for the heterogeneous breast model is Layer 1. This situation affected the energy increase process of primary X-rays in terms of transmission. Figure 5 demonstrates that the energy decrease trend in the first layer as a result of each subsequent energy increase is less significant than the increase trend in the second and third layers. This is because of the variation in density between the layers caused by the GF factors (11). After evaluating each layer separately, the total amount of energy absorbed by the three layers for each energy value is determined and shown in Figure 6 as a function of increasing energy. As shown in Figure 6, the rising trends in the total amount of energy absorbed by the three layers between 26 keV and 30 keV and between 30 keV and 36 keV differ. Low energy X-rays tend to release their energy to the first, second, and third layers, respectively, in the first energy range (26–30 keV). However, although this tendency may also be seen in the second energy range, it is less prominent compared to the first energy range (i.e., 26–30 keV). Although this demonstrates that X-rays with energy over 30 keV have absorption potentials in three layers, it also demonstrates that they have a higher tendency to pass through the third layer and reach the image receptor. As noted in the previous sections, the improvement in transmission properties permits X-rays to travel through a material while maintaining their energy. This is evident when observing the rising trend after 30 keV. Consequently, one can say that the energy-dependent variations in the quantity of energy absorbed in the three layers are maximal in the 26–30 keV energy range. In the meanwhile, the energy amounts absorbed in each of the three layers were computed over the energy range of 26 to 36 keV, and an average value was obtained. Figure 7 is obtained by averaging the results in each of the three layers for each energy value in the examined energy range. The first layer absorbed an average of 52.97 percent of the main X-rays, followed by the second layer with 31.32 percent and the third layer with 15.72 percent. The variation in the quantity of energy absorbed in the body phantom (see Figure 4), modeled behind the three-layered breast phantom was investigated as a function of increasing energy. Figure 8 illustrates the amount of energy absorbed by the body phantom as a function of the increasing energy values. The quantity of energy absorbed by the body phantom increases proportionally to the rise in energy. This tendency, on the other hand, did not change from layer to layer, but rather followed a very consistent rising trend. This condition may be explained by the possibility of scattering from the first, second, and third layers to affect the modeled body phantom. In other words, the inverse ratio of energy release resulting from an increase in transmission found in the layers is non-existent here. This is because the modeled body phantom is always in touch with the heterogeneous three-layered breast phantom. This finding indicates that high energy usage may result in a constant rise in the quantity of dose administered during mammographic examinations in areas other than the examination area. Considering the dose exposures and risks of the chest wall and, therefore, the thoracic region, which is in main contact with the breast tissue during mammography operations, the need of dose optimization may be highlighted once again.

## 4. Conclusion

Dose distribution assessments for mammography operations are becoming increasingly essential. This is due to the fact that breast cancer has one of the highest fatality rates among women. The use of low-energy X-rays enables the very important early detection of this type of cancer. The usage of X-rays, which is a requirement and a necessity of this process, strongly drives the scientific community to perform various types of research on dosimetry procedures. Usually, mammography dosimetry techniques lack experimental feasibility. Thus, sophisticated Monte Carlo simulation approaches, such as EGSnrc, MCNPX, Geant4, Penelope, and codes that simulate radiation transport events are utilized. The purpose of this research was to extend the first phase investigations for the three-layer heterogeneous breast model given in the literature, to address thoroughly some unstudied situations, and to offer data as a continuation of the literature. The scientific community was given the following recommendations based on the findings of the investigation.

- The diverse distribution of glands has a significant impact on the quantity of energy received by the various breast layers.
- In layers with a low glandular ratio, low-energy primary X-ray penetrability is highest. In response to an increase in energy, the absorption in layers with a low glandular ratio decrease.
- This results in the X-rays releasing their energy in the bottom layers.
- Additionally, the increase in energy increases the quantity of energy absorbed by the tissues around the breast.
- The MCNPX code is an important tool that can be used in dosimetry for mammography procedures with the verification it presents together with the values given in standard reports.

In the future, the research group intends to examine the three-layer heterogeneous breast model from other perspectives, such as function of compression level and point distributions in each layer as well as body phantom. Finally, it should be highlighted that further dosimetry studies with such detailed phantoms may benefit the scientific community by giving more comprehensive information on dose reduction, advanced radiation protection strategies, and the assessment of protective materials.

## Data availability statement

The raw data supporting the conclusions of this article will be made available by the authors, without undue reservation.

## Author contributions

GA and ER: writing and calculations. WE: writing and literature review. GK and AE: calculations and visualization. HZ: writing and data analysis. HT: supervision, writing, calculations, and study concept. All authors contributed to the article and approved the submitted version.
